# Oleuropein-Rich Leaf Extract as a Broad Inhibitor of Tumour and Macrophage iNOS in an Apc Mutant Rat Model

**DOI:** 10.3390/antiox10101577

**Published:** 2021-10-06

**Authors:** Jessica Ruzzolini, Sofia Chioccioli, Noemi Monaco, Silvia Peppicelli, Elena Andreucci, Silvia Urciuoli, Annalisa Romani, Cristina Luceri, Katia Tortora, Lido Calorini, Giovanna Caderni, Chiara Nediani, Francesca Bianchini

**Affiliations:** 1 Department of Experimental and Clinical Biomedical Sciences “Mario Serio”, University of Florence, 50134 Florence, Italy; jessica.ruzzolini@unifi.it (J.R.); noemi.monaco@unifi.it (N.M.); silvia.peppicelli@unifi.it (S.P.); e.andreucci@unifi.it (E.A.); lido.calorini@unifi.it (L.C.); 2 NEUROFARBA Department of Neurosciences, Psychology, Drug Research and Child Health, Pharmacology and Toxicology Section, University of Florence, 50139 Florence, Italy; sofia.chiccoli@unifi.it (S.C.); cristina.luceri@unifi.it (C.L.); katia.tortora@unifi.it (K.T.); giovanna.caderni@unifi.it (G.C.); 3 PHYTOLAB (Pharmaceutical, Cosmetic, Food Supplement Technology and Analysis)-DiSIA, Department of Statistics, Informatics, Applications “Giuseppe Parenti”, Scientific and Technological Pole, University of Florence, 50019 Sesto, Italy; silvia.urciuoli@unifi.it (S.U.); annalisa.romani@unifi.it (A.R.); 4 Center of Excellence for Research, Transfer and High Education DenoTHE, School of Medicine, University of Florence, 50134 Florence, Italy

**Keywords:** oleuropein, colon tumours, PIRC rats, activated macrophages, chronic inflammation, inducible nitric oxide synthetase (iNOS), cyclooxygenase-2 (COX-2), nitric oxide (NO)

## Abstract

Oleuropein, the major compound found in olive leaves, has been reported to exert numerous pharmacological properties, including anti-inflammatory, anti-diabetic and anti-cancer effects. The purpose of this study was to evaluate, for the first time, the effect of oleuropein-rich leaf extracts (ORLE) in already-developed colon tumours arising in *Apc* (adenomatous polyposis coli) mutated PIRC rats (F344/NTac-Ap^cam1137^). Here, we were able to investigate in parallel the anti-cancer effect of ORLE, both in vivo and in vitro, and its anti-inflammatory effect on macrophages, representing a critical and abundant population in most solid tumour microenvironment. We found that in vivo ORLE treatment promoted apoptosis and attenuated iNOS activity both in colon tumours as in peritoneal macrophages of PIRC rats. We this confirmed in vitro using primary RAW264.7 cells: ORLE reduced iNOS activity in parallel with COX-2 and pro-inflammatory cytokines, such as IL-1β, IL-6 and TGF-β. These findings suggest that ORLE possess a strong anti-inflammatory activity, which could be crucial for dampening the pro-tumourigenic activity elicited by a chronic inflammatory state generated by either tumour cells or tumour-associated macrophages.

## 1. Introduction

Cancer is currently the second leading cause of death worldwide and highly efficient anti-cancer drugs are currently used to counteract the uncontrolled proliferative activity of neoplastic cells. The effectiveness of most chemotherapeutic agents is accompanied by systemic toxicity, since anti-cancer agents discriminate poorly between normal and cancerous cells. In addition, the efficacy of these treatments is still limited, due to the adverse side effects and the frequent development of resistance.

Among alternative therapies for cancer treatment, there is a growing interest in the anti-cancer action of natural substances, that are non-toxic, affordable, readily accessible, and, some of which, present in large amounts in byproducts from agro-food chains [1].

Natural products are well-established to have pharmacological or biological activities that can be of therapeutic benefits for cancer therapy. Accumulating evidence has revealed that natural products can modulate a series of key signalling pathways displaying therapeutic effects, such as pro-apoptotic, anti-proliferative, anti-angiogenic effects, in different types of human cancers [2]. More recently the use of natural compounds as differentiation inducing agents leading maturation of low differentiated cancer cells rendering them less aggressive and more sensitive to conventional treatments has emerged [3].

The beneficial effects of olive leaves or different preparations (e.g., infusions, extracts) have a several-century-long tradition and have been used for the treatment or to alleviate the symptoms of many diseases (such as diabetes mellitus, arterial hypertension, and bronchial asthma), and are currently contemplated in the Ph. Eur. 5 pharmacopoeia [4].

In particular, *Olea europaea *L. leaves are rich in oleuropein (Ole), a secoiridoid compound that exhibits a wide range of anti-oxidant, anti-inflammatory, anti-diabetic, neuro- and cardio-protective, anti-microbic and immunomodulatory activities [5,6,7,8]. Recently, preclinical studies have provided convincing evidence that Ole has, also, peculiar properties as autophagic, and pro-apoptotic inducer and amyloid fibril growth inhibitor [9,10,11,12,13]. In our experience, we found that Ole might exert an anti-cancer activity, alone or in combination with conventional treatments, through different mechanism in different cancer cell lines [13,14].

Colorectal cancer (CRC), one of most common type of cancer in the Western world of both men and women [15], is one of the solid tumours that may take advantage of a nutritional intervention. Indeed, in addition to a complex genetic susceptibility, the key environmental factors for colon cancer include the diet. Preclinical evidences have demonstrated that olive oil-derived substances have a beneficial effect against colorectal cancer through the modulation of gut microbiota composition or activity [16]. In particular, Ole was able to reduce crypt dysplasia in a rat short-term colon carcinogenesis experiment [17] and to show protective effects in colitis-associated CRC in mice, suggesting, together with the results obtained in cancer cells in vitro, that this molecule may decrease colon tumorigenesis. Whether these protective effects can be extended also to already-developed colon tumours is not known.

Based on these considerations, the aim of the present study is to explore, for the first time, whether oleuropein-rich leaf extracts (ORLE), exerts anti-tumoural and anti-inflammatory activity in colon tumours and peritoneal activated macrophages of PIRC rats carrying a heterozygous germline mutation in the *Apc* gene. The *APC* mutation is the first event triggering colon carcinogenesis both in the majority of sporadic cases and in familial adenomatous polyposis (FAP) syndrome, a hereditary form of colon cancer [18]. Accordingly, PIRC rat spontaneously develops multiple tumours in the colon and small intestine, thus standing as a robust model to study the protective effect of ORLE, derived from olive leaves, on colon cancer progression.

We found that an ORLE enriched diet reduces cell proliferation and increases cell apoptosis in tumours and reduces nitric oxide synthase (iNOS) in colon tumour lesions and peritoneal macrophages of PIRC rats. We confirm that ORLE inhibits the pro-inflammatory features of activated murine macrophages through the reduction of iNOS, cyclooxygenase-2 (COX-2), interleukin (IL)-1β, IL-6 and TGF-β expression, both in acute as in a chronic exposure. We suggest that an ORLE-enriched diet contributes to switching-off the pro-inflammatory signal released either by tumour cells or by inflammatory cells of tumour microenvironment critical for colon cancer progression.

## 2. Materials and Methods

### 2.1. Olive Leaf Extract’s Preparation and Toxicity

Organic olive (Leccino cultivar) leaves were harvested in Tuscany (Vinci, Florence, Italy) and immediately processed to obtain a powder extract rich in active compounds, as previously described in Romani et al. [19]. The characterization of the minor polar compounds and the phenolic profile of olive leaves extract was carried out by HPLC-DAD-MS (high-performance liquid chromatography coupled with diode-array detection and mass spectrometry). The total polyphenol content of dry extract is about 400 mg/g, of which oleuropein was about 379 mg/g. For in vitro experiments ORLE was reconstituted to a final concentration of 14 mM in PBS.

### 2.2. Cell Lines and Culture Conditions

HCT-116, colorectal carcinoma cells were purchased from European Collection of Authenticated Cell Cultures (ECACC, Porton Down, SP4 0JG Salisbury, UK). The murine macrophage RAW 264.7 cell line was purchased from the American Type Culture Collection (ATCC, Manassas, VA, USA. Cells were cultured in Dulbecco’s Modified Eagle Medium high glucose (DMEM 4500, EuroClone, Milan, Italy) supplemented with 10% fetal bovine serum (FBS, EuroClone) and maintained at 37 °C in humidified atmosphere containing 90% air and 10% CO_2_ and they harvested from subconfluent cultures by incubation with a trypsin-EDTA solution (EuroClone) and propagated every three days. Viability of the cells was determined by trypan blue exclusion test. Cultures were periodically monitored for mycoplasma contamination using Chen’s fluorochrome test. HCT116 or RAW cells were exposed for 24 h or 72 h to 50μM ORLE in complete medium according to different experimental procedures. This concentration was tested on colon and macrophage cell lines in preliminary experiments and chosen because resulted non-toxic (see Figure 3a).

### 2.3. MTT Assay

HCT116 cell viability was assessed using MTT (3-(4,5-dimethylthiazol-2-yl)-2,5-diphenyltetrazolium bromide) tetrazolium reduction assay (Sigma Aldrich, Milan, Italy) as described in [13]. Cells (2.5 × 10^3^) were plated into 96-multiwell plates in complete medium without red phenol. The ORLE treatment was added to the medium culture at different dose for 72 h. Then the MTT reagent was added to the medium, and plates were incubated at 37 °C. After 2 h, MTT was removed and the blue MTT–formazan product was solubilized with dimethyl sulfoxide (DMSO, Sigma Aldrich). The absorbance of the formazan solution was read at 595 nm using the microplate reader (Bio-Rad, Milan, Italy).

### 2.4. Animal Maintenance and Ex-Vivo Analysis

PIRC rats (F344/NTac-Apcam1137) and wild type (wt) (Fisher F344) rats were originally obtained by the National Institutes of Health (NIH), Rat Resource and Research Center (RRRC) (University of Missouri, Columbia, MO, USA) and bred in Ce.S.A.L. (Housing Center for Experimental Animals of the University of Florence, Florence, Italy) in accordance with the Commission for Animal Experimentation of the Italian Ministry of Health (EU Directive 2010/63/EU for animal experiments), as described [20]; rats were maintained in polyethylene cages and fed with a standard AIN-76 diet (Laboratorio Dottori Piccioni, s.r.l., Gessate MI, Italy). Eight PIRC rats aged 12 months were randomly assigned to the AIN-76 diet (Control group: two males, two females) or to the same diet containing ORLE (2.7 g/kg of diet) (ORLE group: one male, three females) as reported [13]. The number of rats in each treatment was based on the expected number of tumours/animal in which to carry out our analyses (considering tumour apoptosis as primary endpoint). Accordingly, to observe a significant increase in apoptosis (expected increase of at least 60%, as we previously documented for other cancer-preventive plant compounds [21]), we considered that 6–7 tumours/group, as we actually found, would be sufficient, as calculated with an “a priori “analysis [22]. Considering that rats eat about 11 g of diet/day, and a mean body-weight of 300 g, we administered a dose of ORLE of about 100 mg/kg b.w [13]. Rats were euthanized by CO_2_ asphyxia after one week of treatment, in line with the experimental protocol approved by the Commission for Animal Experimentation of the Italian Ministry of Health. The entire colon and small intestine were flushed with saline solution and opened to check for the presence of tumours which were collected and processed for histological procedure and RNA-extraction as reported [20].

Expression of CD68, as a measure of macrophage infiltration, and expression of proliferating cell nuclear antigen (PCNA), as a measure of proliferative activity, were determined in the tumour lesions of PIRC rats fed with different diets. Longitudinal colon sections (4 µm) were mounted on electrostatic-treated slides (Superfrost^®^ Plus, Medite, Wollenweberstrasse 12 31303 Burgdorf Germany) and processed as described [23] using as primary antibodies: mouse monoclonal antibodies against PCNA (PC-10, Santa Cruz, CA, USA) and rat CD68 (AbD Serotec, Oxford, UK). Both antibodies were diluted in PBS 1:200. CD68 reactivity was quantified as number of labeled cells/areas scored evaluated with the ACT-2U software program (Nikon, Instruments Europe, Badhoevedorp, The Netherlands) connected via a camera to the microscope (Optiphot-2, Nikon, Tokyo, Japan). Evaluation was performed at 400× magnification.

### 2.5. Peritoneal Macrophages Isolation

Macrophage cultures were established from peritoneal exudates collected from rats fed with different diets. Briefly, 20 mL of ice-cold PBS were injected in the peritoneal cavity of the rats and collected immediately. Peritoneal exudates were washed by centrifugation and macrophage monolayers allowed to adhere to plastic dishes in DMEM4500 medium (without phenol red) containing 250 μg/mL bovine serum albumin (BSA), at the density of 125 × 10^3^ cells/cm^2^ in a 24-well dishes. After adhesion, macrophages cultures were washed with PBS and then incubated in DMEM 4500 (w BSA, *w/o* PhR), at 37 °C in a 10% CO_2_ humidified atmosphere and exposed to rIFNγ (50 U/mL) (Immunotools, Friesoythe, Germany) and LPS (10 ng/mL) (Sigma) [24] for 48 h.

### 2.6. Mucosal Samples Collection and RT-PCR Analysis

Mucosal samples were collected in RNAlater and stored at −80 °C until extraction of nucleic acids. DNA quality was assessed by gel electrophoresis and spectrophotometry, measuring OD 260/280.

### 2.7. Determination of Apoptosis

Apoptosis was evaluated in histological sections (4 μm thick) of tumours stained with hematoxylin eosin as recommended by Femia et al. [25], determining cells with the following characteristics of apoptosis: cell shrinkage, loss of normal contact with the adjacent cells of the crypt, chromatin condensation, or formation of round or oval nuclear fragments. Apoptosis was quantified as the number of apoptotic cells/area measured using the ACT-2U software program (Nikon, Instruments Europe) connected via a camera to a microscope (Nikon Optiphot-2). The evaluation was performed at 1000× magnification.

### 2.8. Nitric Oxide Assay

NO concentration was measured in the culture medium of rat peritoneal macrophages or RAW cells using the Griess reaction. Namely, NO production was measured in rat peritoneal macrophages cultures after 48 h of an in vitro treatment with IFNγ and LPS, while in RAW264.7 cells (1.6 × 10^5^ cells/well) was measured after 24 h treatment with 1 μg/mL LPS [26]. In particular, RAW264.7 cells were exposed for 24 h to a co-treatment with LPS and 50 μM ORLE, or for 72 h to 50 μM ORLE pre-treatment and a sequential 24 h treatment with LPS. Briefly, 100 μL of cell culture medium from RAW264.7 cells or 250 μL from peritoneal macrophages cultures were mixed with an equal volume of Griess reagent (1% sulfanilamide, 0.1% N-1-naphthalenediamine dihydrochloride, and 2.5% H_3_PO_4_) and transferred to 96-well plates. Plates were incubated at room temperature for 10 min. Then the absorbance was measured at 540 nm in a microplate reader (BioTek, Winooski, VT, USA). The amount of nitrite in the media was calculated from sodium nitrite (NaNO_2_) standard curve. Results were normalized to protein concentration. For RAW264.7 cells NO production was expressed referred to LPS as 100%.

### 2.9. Cytofluorimetric Assay of iNOS in HCT116 Cells

The expression of intracellular iNOS in HCT116 cells was assessed by flow cytometry using iNOS monoclonal antibody recommended for the detection of NOS2 of mouse, rat and human origin. HCT116 cells were exposed to a standard medium of a medium containing 50 μM ORLE for 72 h. At the end of the incubation, cells were harvested, fixed in ethanol 70%, permeabilized (Triton-X100, 0.01%), and then stained using anti-human iNOS mouse IgG1 monoclonal antibody (sc-7271, Santa Cruz, 1 μg/10^5^ cells). At the end of the incubation (45′, 4 °C), cells were exposed to PE-labeled goat anti-mouse IgG as secondary antibody (#22549814, Immunotools). Stained cells were analyzed on a fluorescence-activated cell sorting (FACS) flow cytometer (FACScan, Becton Dickinson, BD Biosciences Torreyana Rd - 92121 San Diego, CA, USA).

### 2.10. Western Blotting Analysis

RAW cells were exposed to 1 μg/mL LPS alone, or 50 μM ORLE, or LPS/ORLE in complete medium as previously described. After incubation, cells and supernatants were lysed together and separated using electrophoresis [13]. Cells were washed with ice cold PBS containing 1 μM Na_4_VO_3_, and lysed in 100 μL of cell RIPA lysis buffer (Merck Millipore). PMSF (1 mM final concentration), sodium orthovanadate (100 μM final concentration) and protease inhibitor cocktail set III (from Sigma-Aldrich), have been added to RIPA buffer. Aliquots of supernatants containing equal amounts of protein (40 μg) in Laemmli buffer were separated on Bolt^®®^ Bis-Tris Plus gels 4–12 and fractionated proteins were transferred to a PVDF membrane using iBlot 2 system (Life Technologies, Carlsbad, CA, USA). Membranes were blocked for 1 h (RT) using Odyssey blocking buffer. Subsequently, the membranes were probed (4 °C O/N) with primary antibodies. The primary antibodies were diluted 1:1000 in a solution of 1:1 Odyssey blocking buffer/T-PBS buffer. The following antibodies were used: COX-2 rabbit anti h/m/r mAb (#4842S, Cell Signaling, Danvers, MA, USA, 74 kDa), iNOS rabbit anti mouse mAb (#13120S, Cell Signaling, 130 kDa), and IL-1βRI rabbit anti mouse mAb (sc-689, Santa Cruz, 80 kDa). The membranes were washed in T-PBS buffer and incubated for 1 h (RT) with goat anti-rabbit IgG Alexa Flour 750 antibody or with goat anti-mouse IgG Alexa Fluor 680 antibody (Invitrogen, Waltham, MA, USA, 1:10000). Bands were then visualized using Odyssey Infrared Imaging System (LI-COR^®®^ Bioscience, Lincoln, NE, USA). The vinculin rabbit anti h/m/r/mk mAb (#13901, Cell Signaling, 124 kDa) (1:1000) and anti h/m/r/mk mAb α-tubulin (#3873, Cell Signaling, 52 kDa) were used to assess equal amount of protein loaded in each lane.

### 2.11. RNA Extraction and Quantitative PCR

Total RNA from rat tumours or RAW264.7 cells was extracted using the Trizol reagent. The concentration and purity of RNA samples were determined using the 260 to 280 ratio (A260/A280) and the 260 to 230 ratio (A260/A230) readings of an ultraviolet spectrophotometer. The first-strand cDNA was synthesized, from 1 μg of total RNA, using the iScript cDNA Synthesis Kit (#1708891, BioRad, Hercules, CA, USA) following the provided instructions. Quantitative real-time polymerase chain reaction (RT-PCR) was carried out using SYBR Green PCR Master Mix (#4309155, Applied Biosystems, Waltham, MA, USA). The results were analyzed on the (BioRad CFX96 qPCR Instrument). Values were normalized to 18S, and all the results were obtained from at least three experiments independently. The sequences of primers used in this study are listed in Table 1.

### 2.12. Statistical Analysis

All data were obtained based on at least on three independent experiments and expressed as mean ± SEM. Statistical analysis between two groups was performed using unpaired Student’s *t*-tests. When comparing three or more groups for one or two independent variables one-way or two-way analysis of variance (ANOVA) were performed followed by Tukey’s post hoc test. P-values were calculated using GraphPad Prism version 6.04 for Windows (GraphPad Software, La Jolla, CA, USA) and are provided in the figure legends. Band intensities in Western blot analysis were quantified using the computer-based ImageJ software.

## 3. Results

### 3.1. In Vivo Effect of ORLE on PIRC Rats

**Figure 1:**
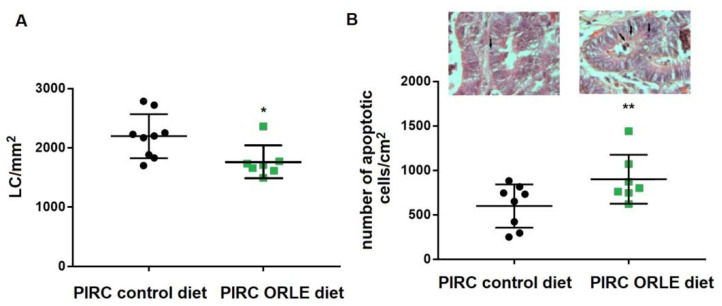
(**A**) Proliferative activity ((PCNA-Labelled cells (LC))/mm^2^) in colon tumours), (**B**) apoptosis (apoptotic cells/mm^2^) in colon tumours) in PIRC rats fed with different diets determined as described in the Methods section. Representative images of either group were shown (arrows indicate apoptotic cells; original magnification 1000×). Statistical significances were determined using two-tailed unpaired student’s *t*-test corresponding to * *p* = 0.022 and ** *p* = 0.041.

One-year old PIRC rats with consolidated colon tumours were fed for one week with ORLE diet or standard diet. At the end of treatment, no differences in body weight were found between the two groups, indicating an unchanged caloric intake. At sacrifice, colon tumours were present in both controls and ORLE treated groups and were processed and analyzed for cell proliferation and apoptosis level. ORLE induced a significant reduction in cell proliferation and augmented the levels of apoptotic bodies in the tumour lesions of PIRC rats, when compared to those of control group fed with a standard diet (Figure 1a).

**Figure 2:**
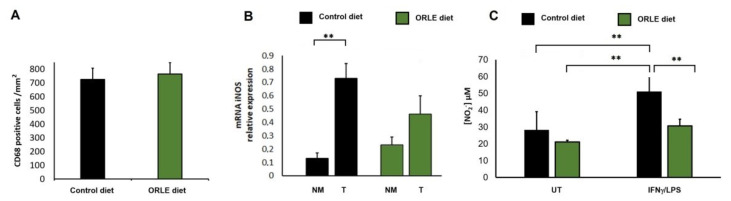
Effect of ORLE on rat macrophages. (**A**) CD-68 expression (positive cells/mm^2^) in colon tumours from PIRC rats fed with a control diet (black column) or a diet enriched in ORLE (green column). (**B**) iNOS expression in colon tumours (T) from PIRC rats fed with a control diet (black columns) or a diet enriched in ORLE (green columns) compared to iNOS expression in normal mucosa (NM). Data are expressed as means + SEM; two-way-ANOVA of the data shows a statistical significance (** *p* = 0.0016) for the effect of tissue (i.e., tumour expression being higher than that in the normal mucosa), while dietary treatment was not significant. Post-hoc analysis of the differences between different groups (Tukey’s multiple comparisons test) shows a significant difference between NM and tumours in the Control diet (** *p* = 0.0047), while the difference between NM and tumours in the ORLE diet is not significant. (**C**) Effect of ORLE on NO production by peritoneal macrophages recovered from tumour bearing rats fed a control diet (black columns) or a diet enriched in ORLE (green columns) and untreated or exposed, in vitro, to IFNγ/LPS. Two-way-ANOVA of the data shows a statistical significance for the effect of ORLE diet (*p* = 0.0001), and for the effect of treatment (*p* = 0.0002). Post-hoc analysis of the differences between different groups (Tukey’s) shows a significant difference as indicated by the asterisks (*p* < 0.001).

To explore the in vivo effect of ORLE, we also evaluated the response of ORLE enriched diet on macrophages. Macrophage recruitment to neoplastic mucosa was determined with the evaluation of CD68 antigen expression in the colon tumours. As showed in Figure 2a, ORLE intake did not alter the number of macrophages infiltrating the lesions. In addition, as NO is often associated with cancer aggressiveness, we determined mRNA level of iNOS in the colon tumours and compared to that expressed in normal mucosa of the same rats. We found that iNOS expression was significantly augmented in tumour lesions compared to their normal mucosa, while in rats fed with an ORLE diet, iNOS mRNA over-expression was not significant compared to their normal mucosa (Figure 2b). The effect of the ORLE diet was also explored in peritoneal macrophages collected upon the sacrifice of rats fed with the two diets. In particular, we examined the ex-vivo NO production by collected macrophages after in vitro treatment with IFNγ and LPS. Macrophages recovered from PIRC rats fed with ORLE diet were unresponsive to the exposure to IFNγ/LPS. Indeed, the release of NO was comparable to that of relative untreated macrophages. Conversely, peritoneal macrophages collected from PIRC rats fed with a control diet were extremely responsive to the exposure to IFNγ/LPS. Indeed, the NO release was remarkably increased compared to relative untreated macrophages (Figure 2c).

Finally, since a reduction of iNOS mRNA was observed in rat colon tumours, we aimed to determine whether the expression of iNOS protein by colon cancer cells changed after the treatment with ORLE. By using flow cytometry analysis, we found a decreased iNOS protein expression in HCT116 cells treated with 50 μM ORLE for 72 h.

**Figure 3:**
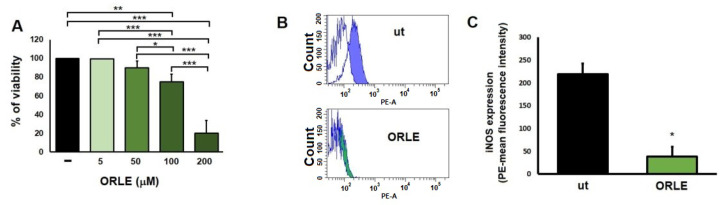
MTT assay of HTC116 cells exposed for 72 h to different concentration of ORLE (**A**). Data are expressed as means ± SEM of the percentage of viability and are representative of three independent experiments (n = 4). Statistical evaluation of the effect of ORLE on tumour cell viability was analyzed by one-way ANOVA with post-hoc Tukey’s test, * *p* < 0.001, ** *p* < 0.0001, and *** *p* < 0.00001. (**B**) Inhibition of intracellular protein expression of iNOS in HCT116 cells. Representative histogram plots of untreated HCT116 cells (blu istogram) or HCT116 cells exposed for 72 h to ORLE (50 μM) (green istograms). Coloured istograms represents HCT116 cells exposed to primary and secondary antibody, while white istograms represents cells exposed to secondary antibody only. (**C**) Quantitative analysis of mean florescence intensity. Data were expressed as means ± SEM (n = 3). Statistical significance was determined using two-tailed unpaired student’s *t*-test corresponding to * *p* < 0.0001.

HCT116 cells exposed to 50 μM ORLE for 72 h treatment did not show, compared to untreated cells, a significant reduction of cell viability. Significant reduction on cell viability was found at higher doses (100 μM and 200 μM ORLE), this resistance is probably related to their high aggressiveness (Figure 3a,b).

### 3.2. Effect of ORLE on Primed Murine RAW264.7 Cells

**Figure 4:**
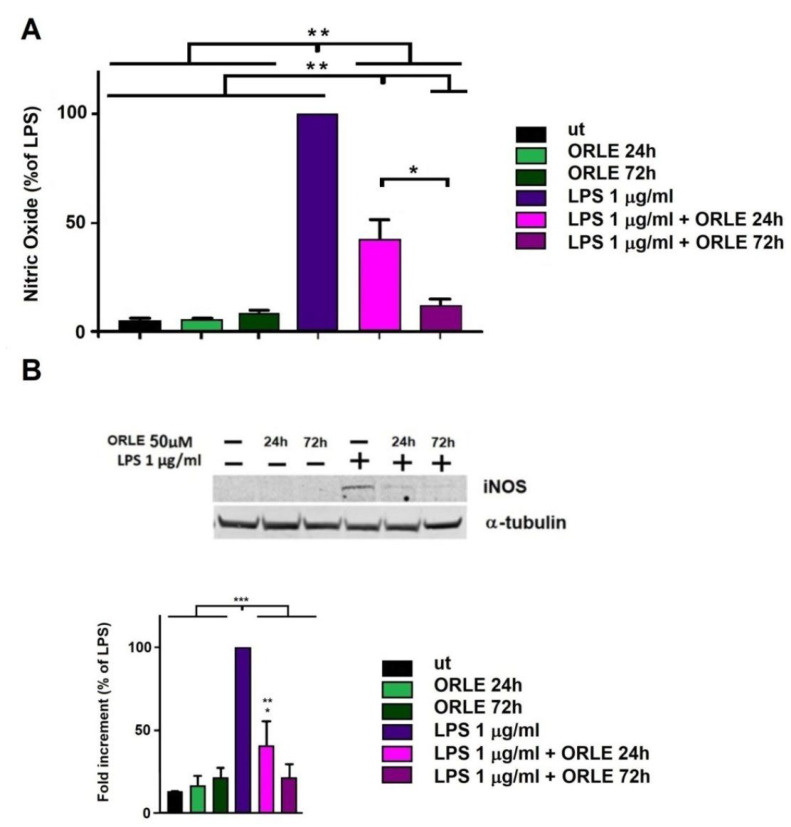
Inhibition of NO production in RAW264.7 macrophages: (**A**) exposed to 50 μM ORLE and 1 μg/mL LPS. Data are expressed as means ± SEM of the percentage of inhibition compared to LPS treated cells. Data are representative of three independent experiments (*n* = 4). Two-way-ANOVA of the data shows a statistical significance for the effect of ORLE and for the effect of LPS (*p* < 0.0001). Post-hoc analysis of the differences between different groups (Tukey’s) shows a significant difference as indicated by the asterisks (* *p* < 0.0001, and ** *p* < 0.01). (**B**) Upper panel, representative Western blot of inducible nitric oxide synthase (iNOS) protein expression, lower panel, densitometric analysis of iNOS expression in RAW.264.7 cells. Data are expressed as means ± SEM of percentage compared to LPS-stimulated cells from at least three independent experiments. Two-way-ANOVA of the data shows a statistical significance for the effect of ORLE and for the effect of LPS (P < 0.001). Post-hoc analysis of the differences between different groups (Tukey’s) shows a significant difference as indicated by the asterisks (* *p* < 0.01, ** *p* <0.05 vs. UT, and *** *p* < 0.0001).

To further explore ORLE effect on macrophages pro-inflammatory activity, we evaluated the release of NO in RAW264.7 cells treated with 50 μM ORLE for 24 h in the presence of LPS (acute exposure) or pretreated with 50 μM ORLE for 72 h (chronic exposure) and next exposed to LPS. NO production, in quiescent RAW264.7 cells, was not affected by the treatment with ORLE, neither after an acute or chronic modality. NO production in RAW264.7 cells activated with ORLE acute exposure was significantly inhibited compared to that measured in absence of it (50% reduction). Interestingly, NO production by LPS-activated macrophages, after ORLE chronic exposure, was strongly inhibited (70% compared to LPS-treated) (Figure 4a). In parallel, ORLE treatment strongly inhibited the expression of iNOS after ORLE acute or chronic exposure (Figure 4B).

**Figure 5:**
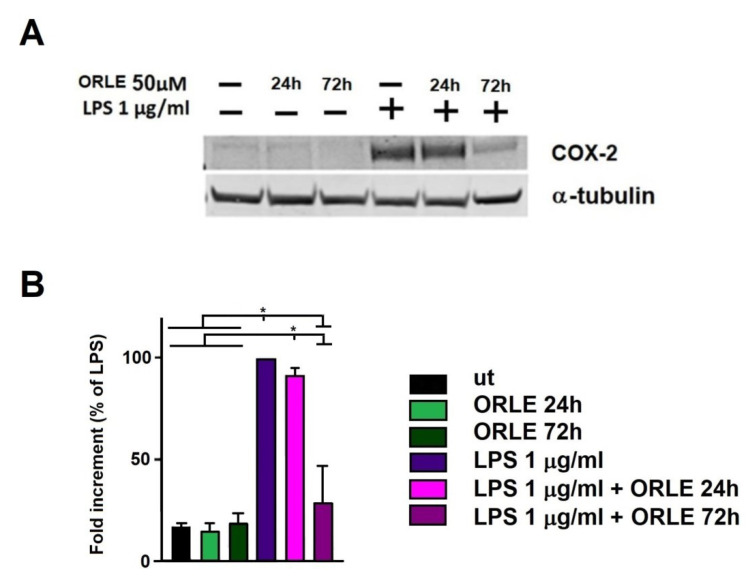
Inhibition of COX-2 expression. (**A**) Representative Western blot of inducible cyclooxygenase-2 (COX-2) protein expression; (**B**) densitometric analysis of COX-2 expression in RAW.264.7 cells. Data are expressed as means ± SEM of percentage compared to LPS-stimulated cells from at least three independent experiments. Two-way-ANOVA of the data shows a statistical significance for the effect of LPS (*p* < 0.005). Post-hoc analysis of the differences between different groups (Tukey’s) shows a significant difference as indicated by the asterisks (* *p* < 0.0001).

Further, ORLE inhibits the expression of COX-2 by an acute treatment and completely abolished it after a chronic exposure (Figure 5). It has to be noted that the expression of iNOS and COX-2 was evaluated on the same set of samples, and, necessarily, the same tubulin reference was used. We hypothesize a possible cooperation of COX-2 with iNOS abrogation during tumour lesion regression. Indeed, over-expression of iNOS may generate reactive mutagenic agents causing as DNA damage or impairment of DNA repair, and COX-2 stimulation leads to sustain tumour growth [27].

**Figure 6:**
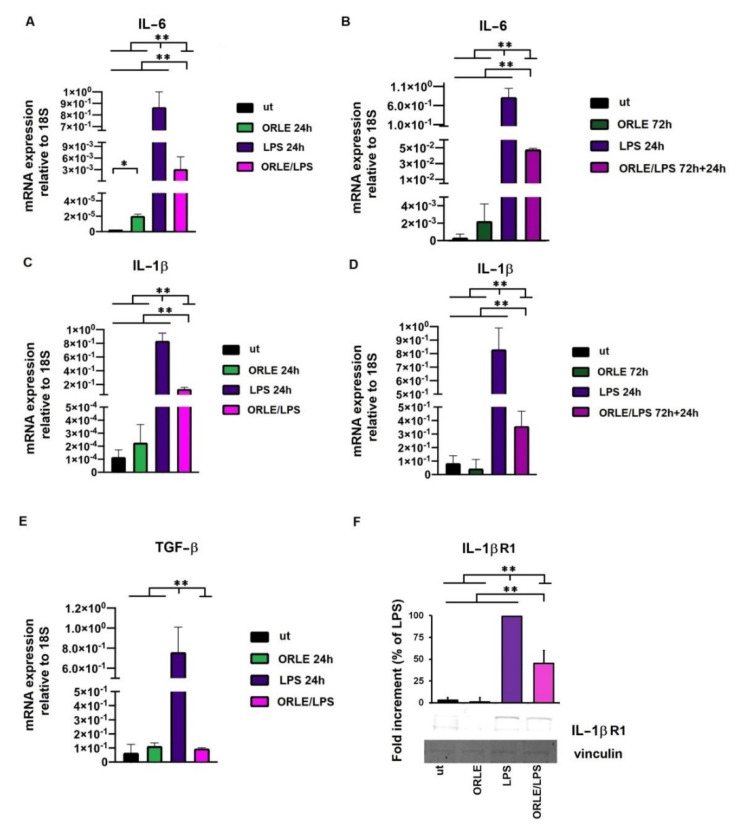
Evaluation by quantitative real-time PCR of IL-6 mRNA (panel (**A**) for ORLE acute, and panel (**B**) for ORLE chronic exposure), and IL-1β mRNA (panel (**C**) for ORLE acute, and panel (**D**) for ORLE chronic exposure) in RAW264.7 cells. Quantitative real-time PCR of TGF-β mRNA in RAW264.7 cells exposed to ORLE acute treatment (**E**). mRNA levels were normalized to 18S as an endogenous control. Two-way-ANOVA of the data shows a statistical significance for the effect of ORLE treatment (*p*: 0.001), and for the effect of LPS (P: 0.002). Post-hoc analysis of the differences between different groups (Tukey’s) shows a significant difference as indicated by the asterisks (* *p* < 0.01 and ** *p* < 0.0001). IL-1R1 protein expression and relative densitometric analysis in RAW264.7 cells exposed to ORLE acute treatment (**F**). Data are expressed as reduction relative to LPS. Western blot images are representative of at least three independent experiments. Two-way-ANOVA of the data shows a statistical significance for the effect of ORLE treatment, and for the effect of LPS (*p*: 0.001). Post-hoc analysis of the differences between different groups (Tukey’s) shows a significant difference as indicated by the asterisks (** *p* < 0.0001).

The evaluation of anti-inflammatory effect of ORLE, either acutely or chronically, on RAW 264.7 macrophages exposed to LPS was finally explored through the analysis of the relative mRNA expression of IL-1β and IL-6 cytokines and TGF-β. The results indicated that ORLE decreased the mRNA expression of IL-1β, and IL-6, in LPS-induced RAW264.7 cells in time-dependent manner, the more prolonged was the exposure to ORLE, the more significant was the inhibitory effect (Figure 6a,b). In addition to this inhibitory effect, ORLE promotes, in an acute exposure, a reduction of IL-1βR protein expression and a reduction of mRNA expression for TGF-β. (Figure 6c,d).

## 4. Discussion

The anti-inflammatory properties of phenolic secoiridoids have been recognized since a long time, and the reduction of oxidative stress and inflammatory cells recruitment, have been clearly demonstrated [28]. Ole is one of the most intriguing members of secoiridoids family, and is able to dampen systemic inflammation through the modulation of pro-inflammatory cell recruitment [29,30]. Given the close correlation between the perseverance of chronic inflammation and tumour progression, Ole and the other phenolic compounds have been studied for their beneficial effect on different models of in vitro and in vivo cancer progression.

The aim of the present study is to evaluate, for the first time, the impact of an ORLE-enriched diet, in an animal model with already-developed colon tumours, exploring its effects on different characteristics of colon cancer and associated systemic inflammation [31]. We focused our interest on in vivo model of colon cancer, using the PIRC rats that spontaneously develop tumours in the colon [32,33]. We choose one-week treatment of ORLE-enriched diet equivalent to the consumption of a Ole dose (100 mg/kg of ORLE) according to previous studies [13,17], to better highlight whether this low-dose treatment might exert a beneficial effect against established cancer lesions and local and systemic inflammation. Secondly, we confirm the anti-inflammation activity of ORLE in murine activated peritoneal macrophages.

The novelty of our study is that one year old PIRC rats, bearing consolidated tumours, were fed with ORLE-enriched diet for a short period of time (one week). On the contrary, previous studies explored the chemoprevention effects of Ole/ORLE during the first phases of the development of the tumours [17,34]. We focused our attention on one of the key mediators released in tumour microenvironment, namely NO. Accordingly, it is well known that the expression of inducible NO synthase in cancer correlates with a patient poor prognosis [35]. However, it should be considered that, due to the highly sophisticated network of interactions, in which the released NO is involved in tumor microenvironment, the role of NO in colon cancer progression remains controversial [36,37]. We demonstrated that ORLE-enriched diet decreased cell proliferation and increased apoptosis in colon tumours in vivo; we also documented that ORLE diet was able to counteract the tumour-associated iNOS over-expression present in the tumours of control rats.

It has to be noted that peritoneal macrophages not only monitor and maintain local homeostasis, but also play the role of sentinel cells against threats such as infections, tissue damages, and tumours [38]. Thus, in some instances, the evaluation of peritoneal macrophages activation could be used as a measure of local and systemic responsiveness to pro-inflammatory stimuli [39]. We found that ORLE-enriched diet dampened the pro-inflammatory behavior of peritoneal macrophages from tumour-bearing rats. Indeed, one-week exposure to ORLE-enriched diet was sufficient to clearly reduce peritoneal macrophages responsiveness to the in vitro treatment with IFNγ/LPS. Thereafter, we demonstrate that ORLE was also effective in reducing in vitro iNOS expression in human adenocarcinoma cells. It has to be noted that we observed an in vitro anti-proliferative effect on colon carcinoma cells only at higher ORLE concentrations. Similar results have been found by others using ORLE [13] or Ole at higher concentrations [40,41].

The upregulation of iNOS and the elevated NO release is an unavoidable aspect of tumour microenvironment. The NO unpaired electron rapidly reacts with other radical species present in the tumour microenvironment driving to an increased DNA damage and the acquisition of additional mutations on surrounding tumor cells contributing in the maintenance of an aggressive tumour phenotype [42,43,44].

Further, in colon cancer the release of cytokines as IL-1β, IL-6 and the activation of COX-2, collectively, support neoplastic transformation and malignant progression [45,46]. Thus, we investigated whether ORLE treatment might modulate COX-2 and cytokine release of activated macrophages. There is an intense debate regarding the role of tumour-associated macrophages and cancer outcomes, mostly due to the highly plastic behavior of these immune cell population [47,48]. Despite this, clinical evidence suggests that, in colon cancer, the presence of a rich macrophage infiltrate accounts for a better prognosis [47]. Interestingly, we found that the number of macrophages infiltrating tumour lesions of ORLE enriched diet fed rats was similar to that of macrophages infiltrating tumour lesions in rats fed a regular diet. Conversely, we found that ORLE treatment significantly inhibited LPS activated macrophages, in term of NO release and iNOS expression, consistent with our in vivo results and other studies [26]. In addition to this, we found that COX-2 expression in LPS-activated macrophages was completely abolished after ORLE chronic exposure. We also found that ORLE treatment significantly downregulates LPS-macrophage activation in term of IL-1β, IL-6, and TGF-β mRNA expression. These cytokines and growth factor strongly cooperate in the maintenance of a pro-inflammatory and toxic microenvironment, that correlates with disease progression and drug resistance [39,49,50,51].

## 5. Conclusions

The present study assesses whether one week-low-dose treatment with an ORLE-enriched diet exerts a beneficial effect against established colon cancer lesions of PIRC rats and local and systemic inflammation. Although in vivo experiments were performed with a limited number of PIRC rats fed with ORLE, the overall results disclose a significant increase in tumour apoptosis together with a downregulation of proliferation associated with the inhibition of NO and relative pro-inflammatory mediators expressed by tumour cells and inflammatory cells of tumour microenvironment. These findings suggest the possibility to test ORLE as a complementary therapy in combination with standard anti-cancer drugs.

## Figures and Tables

**Table 1:** Primer sequences for qRT-PCR.

Gene Name (Accession nr.)	Forward Sequence (5′-3′)	Reverse Sequence (5′-3′)
**IL-1β** (NM_008361)	CCT GCA GCT GGA GAG TGT GGA	CCC ATC AGA GGC AAG GAG GAA
**IL-6** (NM_031168)	CTT CCA TCC AGT TGC CTT CT	TGC ATC ATC GTT GTT CAT AC
**TGF-β** (NM_011577.2)	GGC TTC TAG TGC TGA CG	GGG TGC TGT TGT ACA AAG
**18S** (NR_003278)	CGC CGC TAG AGG TGA AAT TCT	CGA ACC TCC GAC TTT CGT TCT

## Data Availability

Data is contained within the article.
